# Prophylactic Central Neck Dissection for cN1b Papillary Thyroid Carcinoma: A Systematic Review and Meta-Analysis

**DOI:** 10.3389/fonc.2021.803986

**Published:** 2022-01-14

**Authors:** Xing-qiang Yan, Zhen-zhen Zhang, Wen-jie Yu, Zhao-sheng Ma, Min-long Chen, Bo-jian Xie

**Affiliations:** ^1^ Department of Surgical Oncology, Taizhou Hospital of Zhejiang Province, Wenzhou Medical University, Linhai, China; ^2^ Department of Plastic Surgery, Enze Hospital of Taizhou Enze Medical Center (Group), Luqiao, China

**Keywords:** prophylactic central neck dissection (PCND), papillary thyroid carcinoma (PTC), lateral neck dissection (LND), lateral cervical lymph node metastases, locoregional recurrence, total thyroidectomy (TT)

## Abstract

**Background:**

The value of prophylactic central neck dissection (PCND) for papillary thyroid carcinoma (PTC) with clinically evident lateral cervical lymph node metastases (cN1b) remains unclear. Therefore, a systematic review and meta-analysis was conducted to assess the efficacy and safety of PCND.

**Methods:**

A comprehensive systematic search was conducted on PubMed, Web of Science, Cochrane library and Embase databases up to September 2021 to identify eligible studies. Controlled clinical trials assessing therapeutic effects and safety of PCND for cN1b PTC patients were included. The risk of bias for each cohort study was assessed using the Newcastle-Ottawa Scale (NOS). The primary outcomes were indexes related to the locoregional recurrence (LRR) and surgical complications. Review Manager software V5.4.0 was used for statistical analysis. A fixed effects model was adopted for the data without heterogeneity, otherwise a random effects model was used.

**Results:**

We included 4 retrospective cohort studies, which comprised 483 PTC patients. There was no statistically significant difference in the central neck recurrence (CNR) (10.2% vs. 3.8%, relative risk (RR) = 1.82; 95%CI 0.90–3.67; P = 0.09), lateral neck recurrence (LNR) (5.1% vs. 7.7%, RR = 0.47; 95% CI 0.13–1.74; P = 0.26), and overall recurrence (OR) (18.9% vs. 16.9%, RR = 0.77; 95%CI 0.34–1.76; P = 0.54), between LND + PCND group and LND group. Simultaneously, PCND increased the risk of permanent hypoparathyroidism (11.4% vs. 4.5%, RR = 2.70, 95%CI 1.05–6.94; P = 0.04) and overall complications (17.0% vs. 5.3%, RR = 3.28; 95%CI 1.37–7.86; P = 0.008).

**Conclusions:**

This meta-analysis showed that PCND did not have any advantage in preventing LRR for cN1b PTC. Meanwhile, PCND may result in the increased rate of surgical complications. However, the current evidence is limited and more clinical trials are still needed to further clarify the true role of PCND.

**Systematic Review Registration:**

https://www.crd.york.ac.uk/PROSPERO/, CRD42021281825.

## Introduction

Thyroid cancer is the most common malignant tumor in the endocrine system and head and neck tumors, causing586,000 cases worldwide and ranking 9th in incidence in 2020 (1). Papillary thyroid carcinoma (PTC) accounts for the vast majority of thyroid cancers. Regional lymph node metastases (LNM) are very common in patients with PTC (up to 80%), especially in the central compartment of the neck (2, 3). LNM have been reported in association with a higher rate of locoregional recurrence (LRR) (4). Surgical resection of clinically nodal-positive disease (cN1) in PTC is considered to make improvement to the results of both recurrence and survival. Therefore, it is generally believed that therapeutic cervical lymph node dissection is indicated in PTC patients with cN1. However, the effect on long-term outcome of prophylactic central neck dissection (PCND) in PTC patients without clinically evident nodal metastasis (cN0) remains unclear, and the 2021 National Comprehensive Cancer Network (NCCN) and the 2015 American Thyroid Association (ATA) guidelines do not recommend that routine PCND should be performed in all these PTC patients with cN0 (5, 6).

There is a generally accepted assumption that cervical LNM in PTC patients follow the gradual progression of LNM from the central to lateral compartment and lymph nodes skip metastases to the lateral compartment are present only in a small number of PTC patients (7, 8). Therefore, some surgeons have advocated routine PCND in PTC patients with clinically evident lateral cervical LNM (cN1b) combined with total thyroidectomy (TT) and lateral neck dissection (LND) (9). Moreover, the 2021 NCCN and the 2015 ATA guidelines suggest that PCND should be taken into consideration in PTC patients with cN1b (5, 6). However, this suggestion is relatively weak and based on low quality evidence, and the value of PCND remains unclear.

Surgery in the central compartment of neck may result in some complications. The majority of these complications are injuries of the parathyroid glands and recurrent laryngeal nerves, which will lead to temporary or permanent hypoparathyroidism and hoarseness of voice. As is well known, the extent of initial surgery of thyroid and lymph nodes should be based on evidence of long-term benefits in terms of improvements of local control or survival, while minimizing the risk of complications. Hence, for these PTC patients with cN1b but no clinically evident central compartment LNM on preoperative imaging or intraoperative evaluation, we should weigh the potential benefits of PCND against the risks. This systematic review and meta-analysis was conducted to evaluate the LRR and complications rates of TT + LND versus TT + LND + PCND.

## Methods

### Study Protocol and Registration

**Figure 1:**
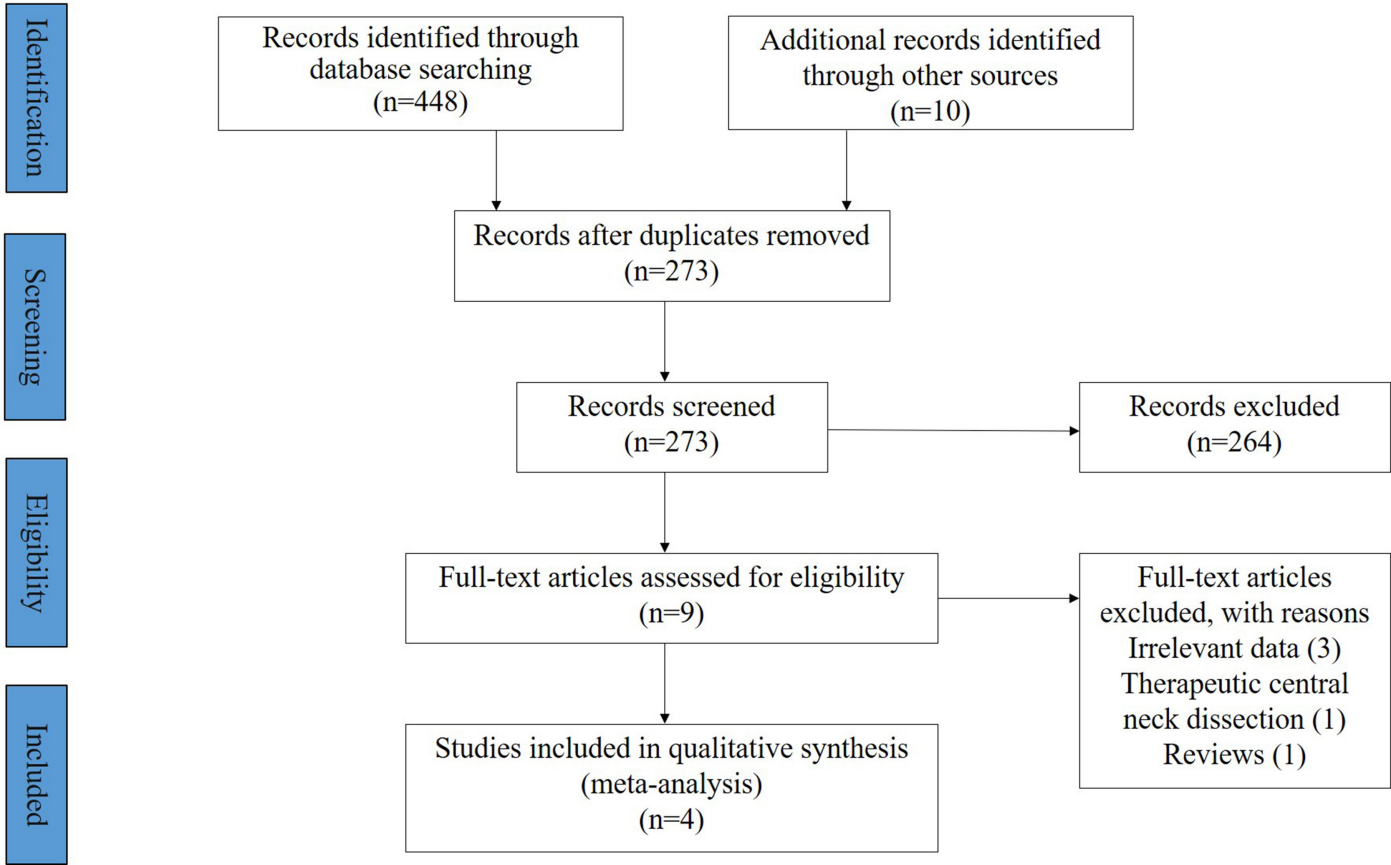
Flowchart of article selection according to PRISMA statement.

This systematic review and meta-analysis was performed in accordance with the guidelines of the Preferred Reporting Items for Systematic Reviews and Meta-Analyses (PRISMA) (**Figure 1**). The study protocol was registered on PROSPERO with No. CRD42021281825.

### Search Strategy

Eligible studies were identified by a comprehensive systematic search on the PubMed, Web of Science, Cochrane library and Embase databases up to September 2021. The search strings used a combination of the terms “papillary thyroid cancer”, “well-differentiated thyroid carcinoma”, “lateral neck dissection”, “elective central neck dissection”, “prophylactic central neck dissection”, and logical word “OR” or “AND”.

### Study Selection

Two investigators independently used search strategies to retrieve study titles and abstracts. The inclusion criteria for the articles were as follows (1): articles published in English, (2) PTC patients diagnosed with lateral compartment LNM, (3) two groups that compared TT + LND with TT + LND + PCND in study. The exclusion criteria were as follows: (1) lack of preoperative assessment of central compartment lymph nodes, (2) preoperative diagnosis of central compartment LNM, and (3) insufficient demographic and clinical data. Letters to editors, case reports, meeting abstracts, review articles and incomplete studies were also excluded. In addition, both investigators also reviewed the reference lists of included articles to identify additional relevant articles. All of the potentially eligible articles were identified, and any disagreements between the two investigators were resolved through a discussion and by consensus with a third investigator.

### Data Extraction and Quality Assessment

Two investigators independently completed extraction of the following data from included articles based on a standardized template: first author, year, country, study design, study period, follow up, sample size, age, sex, tumor size, extrathyroidal extension, incidence of central LNM, radioactive iodine (RAI) treatment, number of LRR, site of LRR, and surgical complications. For cohort studies, quality assessment and analysis of risk of bias were conducted by Y-xQ and Z-zZ using Newcastle-Ottawa Scale (NOS) independently. This scale awards a maximum of nine points. A score of ≥7 is considered to indicate high quality.

### Statistical Analysis

Review Manager software V5.4.0 (The Cochrane Collaboration, 2020) was used for statistical analysis. Heterogeneity among studies was assessed by the heterogeneity test with the I^2^ statistic. I^2^ >50% was considered significant heterogeneity. A fixed effects model was adopted for the data without heterogeneity in the meta-analysis, otherwise a random effects model was used for the significantly heterogeneous data. The difference between the two groups was quantified by the terms of relative risk (RR) along with 95% confidence interval (CI), and P <0.05 was considered to be significance.

## Results

### Study Selection

The detail of the article selection process is described as a flow chart (**Figure 1**). Our search strategy retrieved a total of 458 articles, and 273 unique articles were retained after removing the duplicates. After reviewing the titles and abstracts according to the inclusion and exclusion criteria, 9 potentially eligible articles were obtained and reviewed in full text. Of these 9 articles, 5 were subsequently excluded for various reasons, and finally 4 retrospective cohort studies involving 483 PTC cases were included in the meta-analysis (286 in the LND group and 197 in the LND + PCND group) (10–13).

### Study Characteristics and Quality

The characteristics of these included studies are summarized in **Table 1**. The years of publication of the included articles ranged from 2013 to 2020. Two studies were conducted in the Israel (10, 13), one in the USA (12), and one in Italy (11). All studies underwent a preoperative imaging procedure for central compartment lymph node assessment by ultrasonography, CT scan, or MRI scan, and two studies also performed an intraoperative evaluation. All studies made a comparison of gender ratio, age, tumor size, multifocality, extrathyroidal extension, metastatic lateral cervical lymph nodes, maximum size of metastatic lateral cervical lymph nodes, and RAI treatment between the two groups. Three studies showed a high incidence of central LNM ranging from 70.6 to 87.0%. The follow-up period ranged from 44 to 124 months. Recurrence of disease was determined by cervical imaging evaluation or serum thyroglobulin level and pathologically confirmed (if possible). Quality assessment is shown in **Table 2**. The exposed (patients underwent PCND) and nonexposed cohorts in all included studies were considered good representatives. The main outcomes of interest were LRR and complications. The distribution of preoperative clinical and demographic characteristics was comparable between these two groups, but the status of lateral cervical lymph nodes in each cohort was not compared in the Carmel‐Neiderman et al. study. The assessment of outcome was obtained from laboratory and imaging data. All studies provided the complete follow-up of all patients, but the mean follow-up period was less than 10 years in three studies, which was considered insufficient.

**Table 1:** Detailed characteristics of studies included in the meta-analysis.

Study	Year	Country	Study design	Study period	Follow up (mean, month)	Sample size	Age (mean, year)	Sex (M/F)	Tumor size ≤4cm, n (%)	Extrathyroidal extension, n (%)	Incidence of central LNM, n (%)	Radioactive iodine treatment, n (%)
Trivizki et al.	2013	Israel	RCS	2000–2010	44	LND + PCND: 34	47	17/17	17 (50.0)	13 (38.2)	24 (70.6)	34 (100)
						LND: 17	44	11/6	10 (58.8)	5 (29.4)		17 (100)
Napoli et al.	2020	Italy	RCS	2004–2015	67.9	LND + PCND: 54	42	13/41	49 (90.7)	34 (63.0)	47 (87.0)	54 (100)
						LND: 115	45	45/70	108 (93.9)	68 (59.1)		115 (100)
Harries et al.	2020	USA	RCS	1986–2015	65	LND + PCND: 49	49	19/30	48 (98.0)	/	37 (75.5)	38 (77.6)
						LND: 103	49	46/57	99 (96.1)	/		73 (70.9)
Carmel‐Neiderman et al.	2020	Israel	RCS	1998–2015	124	LND + PCND: 60	48	37/23	/	34 (56.7)	/	59 (98.3)
						LND: 51	53	30/21	/	22 (46.8)		51 (100)

RCS, retrospective cohort study; LND, lateral neck dissection; PCND, prophylactic central neck dissection; LMN, lymph node metastasis.

**Table 2:** Quality assessment of cohort studies.

Study	Representativeness of the exposed cohort	Selection of control	Ascertainment of exposure	Outcome of interest not present at the start of the study	Comparability of controls	Outcome assessment	Sufficient follow-up duration	Adequacy of follow-up	Overall bias
Trivizki et al.	★	★	★	★	★★	★	–	★	8/9
Napoli et al.	★	★	★	★	★★	★	–	★	8/9
Harries et al.	★	★	★	★	★★	★	–	★	8/9
Carmel‐Neiderman et al.	★	★	★	★	★	★	★	★	8/9

The ★ means one point and the ★★ means two points.

### Recurrence

**Figure 2:**
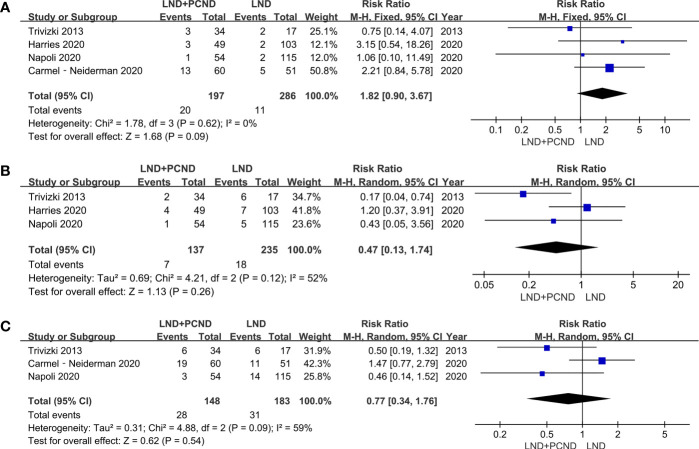
Forest plot showing a meta-analysis of locoregional recurrence (LRR) for included studies. **(A)** Central neck recurrence; **(B)** Lateral neck recurrence; **(C)** overall recurrence.

All studies reported data for central neck recurrence (CNR), 20 patients in the LND + PCND group and 11 patients in the LND group. There was no statistically significant difference between the two groups in the CNR (10.2% vs. 3.8%, RR = 1.82; 95%CI 0.90–3.67; P = 0.09; I^2^ = 0%). Three studies described lateral neck recurrence (LNR) (10–12), 7 patients in the LND + PCND group and 18 patients in the LND group. The rate of LRR in the lateral neck region was also similar between the two groups (5.1% vs. 7.7%, RR = 0.47; 95%CI 0.13–1.74; P = 0.26; I2 = 52%) (10–12). Moreover, we evaluated the overall recurrence (OR) in three studies involving 331 patients (148 received LND + PCND and 183 received LND). A total of 28 patients in total relapsed in the LND + PCND group and 31 patients in the LND group. There was no statistically significant difference between the two groups in OR (18.9% vs. 16.9%, RR = 0.77; 95%CI 0.34–1.76; P = 0.54; I^2^ = 59%) (10, 11, 13). Therefore, the outcomes of this meta-analysis suggested no statistically significant difference between the two groups in LRR and OR (**Table 3** and **Figure 2**).

**Table 3:** Details for recurrence of included studies.

Study	Group	CNR, n (%)	LNR, n (%)	OR, n (%)
Trivizki et al.	LND + PCND	3 (8.8)	2 (5.9)	6 (17.6)
	LND	2 (11.8)	6 (35.3)	6 (35.3)
Napoli et al.	LND + PCND	1 (1.9)	1 (1.9)	3 (5.6)
	LND	2 (1.7)	5 (4.3)	14 (12.2)
Harries et al.	LND + PCND	3 (6.1)	4 (8.2)	/
	LND	2 (1.9)	7 (6.8)	/
Carmel‐Neiderman et al.	LND + PCND	13 (21.7)	/	19 (31.7)
	LND	5 (9.8)	/	11 (21.6)

LND, lateral neck dissection; PCND, prophylactic central neck dissection; CNR, central neck recurrence; LNR, lateral neck recurrence; OR, overall recurrence.

### Surgical Complications

**Figure 3:**
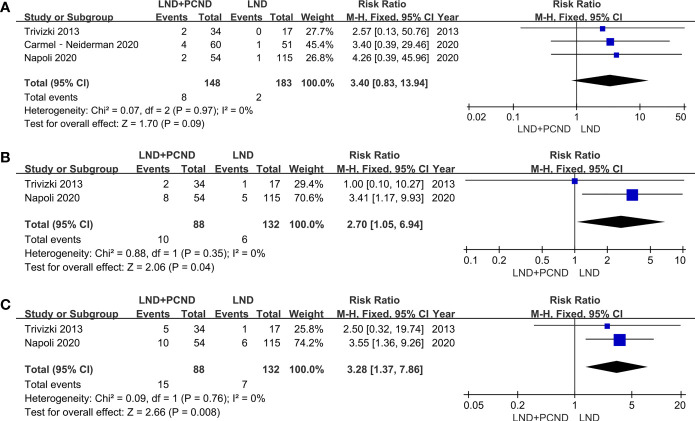
Forest plot showing a meta-analysis of postoperative complications for included studies. **(A)** Permanent laryngeal nerve palsy; **(B)** permanent hypoparathyroidism; **(C)** overall complications.

Data regarding to permanent laryngeal nerve palsy was reported in three studies, 8 patients in the LND + PCND group and 2 patients in the LND group. Prevalence of laryngeal nerve injury in the LND + PCND group and LND group was 5.4% (8/148) and 1.1% (2/183), respectively. Although the incidence of permanent laryngeal nerve palsy in the LND + PCND group was higher than that of the LND group, the outcome of this meta-analysis suggested no statistically significant difference (5.4% vs. 1.1%, RR = 3.40; 95%CI 0.83–13.94; P = 0.09; I2 = 0%). Data of permanent hypoparathyroidism and overall complications was reported in two studies (10, 11). A total of 10 patients presented permanent hypoparathyroidism in the LND + PCND group while 6 patients in the LND group, indicating a higher rate of permanent hypoparathyroidism in the LND + PCND group (11.4% vs. 4.5%, RR = 2.70, 95%CI 1.05–6.94; P = 0.04; I^2^ = 0%). A similar result was obtained for overall complications (17.0% vs. 5.3%, RR = 3.28; 95%CI 1.37–7.86; P = 0.008; I2 = 0%) (**Table 4** and **Figure 3**).

**Table 4:** Details for surgical complications of included studies.

Study	Group	Permanent laryngeal nerve palsy, n (%)	Permanent hypoparathyroidism, n (%)	Overall complications, n (%)
Trivizki et al.	LND + PCND	2 (5.9)	2 (5.9)	5 (14.7)
	LND	0 (0.0)	1 (5.9)	1 (5.9)
Napoli et al.	LND + PCND	2 (3.7)	8 (14.8)	10 (18.5)
	LND	1 (0.9)	5 (4.3)	6 (5.2)
Harries et al.	LND + PCND	/	/	/
	LND	/	/	/
Carmel‐Neiderman et al.	LND + PCND	4 (6.7)	/	/
	LND	1 (2.0)	/	/

LND, lateral neck dissection; PCND, prophylactic central neck dissection.

## Discussion

Nowadays, the management of cervical lymph node dissection in the treatment of PTC is still one of most controversial issues. As everyone knows that therapeutic lymph node dissection for PTC patients will remove all metastatic lymphoid tissue and reduce the LRR rate. However, there is still a lack of sufficient evidence regarding the confirmation of the efficacy and benefit of PCND for PTC patients. Nowadays, the role of PCND in PTC patients with cN0 has been elaborately investigated. Some studies suggested that PCND in cN0 cases would make improvements to locoregional control and disease‐specific survival (14–18). Conversely, other studies suggested that PCND did not improve the long‐term prognosis, but increased the risk of complications (19–24). The difference between the same approach and the results of these studies, may be potentially settled in a prospective randomized controlled trial. However, the ATA investigation revealed that it was not feasible to conduct such a large prospective randomized controlled trial of PCND in PTC patients with cN0 (25). At present, the ATA and NCCN guidelines recommend that PCND should be considered in PTC patients with cN0 with advanced tumor or PTC patients with cN1b (5, 6). However, this recommendation is weak and the evidence is of low quality. The present study showed that a very select group of PTC patients with cN1b but no clinically evident central compartment lymph nodes involvement, might not require routine PCND. The additional procedure does not decrease the rate of LRR and OR, but it is accompanied by complications.

As we all know, regional LNM are very common in PTC patients. Studies have suggested that occult metastases can be found in the central compartment in 40–70% of cases (4, 11, 26). Due to the high rate of occult metastases, PCND may convert many patients from cN0 to pathologic N1a. However, occult metastases do not carry the similar recurrence risk compared with that of clinically detectable macro-metastases (2). The accurate staging information of lymph nodes may be helpful in the selection of adjuvant therapy in PTC patients with cN0, but it is almost useless for these PTC patients with cN1b. These patients will undergo adjuvant RAI treatment and suppression treatment followed surgery in accordance with the ATA guidelines (6). Adjuvant RAI treatment can improve the lymph node failure-free survival in all lymph node categories, and the greatest therapeutic benefits have been observed in patients with cN1b disease (27, 28). Clinically occult lymph node metastasis can also be successfully treated with adjuvant RAI treatment, with an improvement of 10-year lymph node failure-free survival rate from 82.3 to 95% and overall survival rate more than 90% in PTC patients with cN0 (27, 29). Therefore, adjuvant RAI treatment can contain the LRR and might be used as an alternative to PCND.

Additionally, the potential complications of PCND should not be overlooked. In this study, there was a significantly higher rate of permanent hypoparathyroidism in these patients treated with PCND. In fact, it is a quite common phenomenon that PCND leads to a high proportion of both transient and permanent hypoparathyroidism. The reason may be that the inferior parathyroid glands are removed or devascularized unintentionally during the procedure of PCND. Previous literatures also suggest that PTC patients who underwent PCND had a significantly higher incidence rate of permanent hypoparathyroidism compared with those patients managed without PCND (4.11–19.4% VS. 1.95–8%) (24, 30, 31). Although, there was no statistically significant difference in permanent laryngeal nerve palsy by the meta-analysis, the rate of permanent laryngeal nerve palsy following PCND is 3.7–6.7% compared with 0–2.0% in patients managed without PCND. Nowadays, intraoperative laryngeal nerve monitoring has been widely used for reducing the incidence of recurrent laryngeal nerve injury, and some intraoperative parathyroid identification techniques will be helpful to protect the parathyroid in surgery (32, 33). However, the extent of initial surgery should ultimately be determined by oncological benefit.

We acknowledge that there are several limitations in this meta-analysis. Firstly, only 4 articles were included in this meta-analysis and all studies were of retrospective design with small simple size, which limited our ability to draw firm conclusions from the data. Secondly, since PTC may relapse 10 to 20 years after initial treatment, long-term follow-up is necessary to compare the recurrence rate instead of only about 5 years follow-up. Thirdly, the quality of the included studies was not high. Selection bias or other confounding factors might be included and affect the reliability of the final results. Fourthly, because only articles in English were reviewed and included, some non-English articles may have been excluded from the present study.

## Conclusions

In conclusion, this meta-analysis did not demonstrate any advantage in performing PCND in cN1b PTC patients without clinical evidence of central neck involvement for preventing LRR, even in the central region. Furthermore, PCND may result in the increased rate of surgical complications, including a higher rate of permanent hypoparathyroidism and overall complications. We recommend that PCND should not be routinely performed in PTC patients with cN1b, especially for these patients without any clinically evident central neck involvement. However, the current evidence is limited and we need more evidence from multicenter, prospective, randomized, controlled clinical trials to further clarify the true role of PCND in PTC patients with cN1b.

## Data Availability Statement

The original contributions presented in the study are included in the article material. Further inquiries can be directed to the corresponding author.

## Author Contributions

Study conception and design: B-jX and X-qY. Acquisition of data: X-qY, Z-zZ, and W-jY. Analysis and interpretation of data: Z-sM, M-lC and X-qY. Drafting of manuscript: X-qY, Z-zZ. Critical revision of manuscript: B-jX. All authors contributed to the article and approved the submitted version.

## Conflict of Interest

The authors declare that the research was conducted in the absence of any commercial or financial relationships that could be construed as a potential conflict of interest.

## Publisher’s Note

All claims expressed in this article are solely those of the authors and do not necessarily represent those of their affiliated organizations, or those of the publisher, the editors and the reviewers. Any product that may be evaluated in this article, or claim that may be made by its manufacturer, is not guaranteed or endorsed by the publisher.
